# Construction of a hypoxia-derived gene model to predict the prognosis and therapeutic response of head and neck squamous cell carcinoma

**DOI:** 10.1038/s41598-022-17898-2

**Published:** 2022-08-08

**Authors:** Haibin Wang, Lian Zheng

**Affiliations:** Departments of Oral and Maxillofacial Surgery, The First Affiliated Hospital of Zhengzhou University, No. 1 Jianshedong Road, Erqi District, Zhengzhou, 450052 China

**Keywords:** Head and neck cancer, Biomarkers

## Abstract

Head and neck squamous cell carcinoma (HNSCC) ranks as the sixth most common cancer worldwide and has a poor prognosis in the advanced stage. Increasing evidence has shown that hypoxia contributes to genetic alterations that have essential effects on the occurrence and progression of cancers. However, the exact roles hypoxia-related genes play in HNSCC remain unclear. In this study, we downloaded the mRNA expression profiles and clinical data of patients with HNSCC from The Cancer Genome Atlas and Gene Expression Omnibus. Two molecular subtypes were identified based on prognostic hypoxia-related genes using the ConsensusClusterPlus method. ESTIMATE was used to calculate the immune score of each patient. Kyoto Encyclopedia of Genes and Genomes and Gene Ontology were used for functional annotation. A prognostic risk model was generated by Cox regression and least absolute shrinkage and selection operator analysis. We identified two distinct molecular subtypes, cluster 1 and cluster 2, based on 200 hypoxia-related genes. Additionally, we identified three hypoxia-immune subgroups (hypoxia-high/immune-low, hypoxia-low/immune-high, and mixed subgroups). The hypoxia-high/immune-low group had the worst prognosis, while the hypoxia-low/immune-high group had the best prognosis. Patients in the hypoxia-low/immune-high group were more sensitive to anti-PD-L1 treatment and chemotherapy than those in the hypoxia-high/immune-low group. Furthermore, we constructed a prognostic risk model based on the differentially expressed genes between the hypoxia-immune subgroups. The survival analysis and time-dependent ROC analysis results demonstrated the good performance of the established 7-gene signature for predicting HNSCC prognosis. In conclusions, the constructed hypoxia-related model might serve as a promising biomarker for the diagnosis and prognosis of HNSCC, and it could predict immunotherapy and chemotherapy efficacy in HNSCC.

## Introduction

Head and neck squamous cell carcinoma (HNSCC) refers to a group of malignancies that develop from the mucosal epithelium in the larynx, pharynx, and oral cavity and ranks as the sixth most common cancer worldwide^1,2^. Alcohol consumption, tobacco exposure and infection with high-risk human papillomaviruses are the most important risk factors for HNSCC^3^. Although the 5-year survival rate of HNSCC has largely improved, increasing from 55 to 60% over the past three decades^4,5^, most patients are diagnosed at the advanced stage since HNSCC lacks significant patient symptomatology, leading to limited effective treatment. Therefore, there is still a need to better understand the pathogenesis and molecular mechanism of HNSCC and to find novel biomarkers for diagnosis and prognosis.

The tumor microenvironment (TME) is the biological environment of solid tumors. Hypoxia or diminished oxygen availability is a typical feature of the TME^6^, arising from an imbalance between decreased oxygen supply and increased oxygen consumption. Hypoxia in solid tumors changes gradually, contributing to the plasticity and heterogeneity of tumors. Notably, hypoxia contributes to genetic alterations that have essential effects on the occurrence and progression of tumors^7^. Furthermore, the hypoxic microenvironment promotes the aggressive nature of tumor cells and impairs therapeutic efficiency^7–9^ HNSCC tumors are closely correlated with hypoxia, which promotes malignant behaviors (including antiapoptosis, proliferation, invasion, and migration) and is an independent risk factor for HNSCC^10^. In recent years, great attention has been focused on the identification of molecular markers of hypoxia that are potential prognostic and diagnostic biomarkers and promising therapeutic targets in patients with HNSCC^11^. For example, HIF-1a is upregulated in HNSCC cells and positively associated with tumor aggressiveness, enhanced tumor angiogenesis and poor prognosis^12,13^. However, previous studies have been confined to only one or two hypoxia-related genes, while the function and regulatory mechanisms of hypoxia in the TME are complex processes involving multiple genes. Therefore, comprehensive analysis of the various hypoxia-related genes and the relationship between hypoxia and the immune microenvironment of HNSCC is needed to develop novel biomarkers for diagnosis and prognosis.

Here, we collected the gene expression data of hypoxia-related genes and clinical information from The Cancer Genome Atlas (TCGA) and Gene Expression Omnibus (GEO) databases and constructed molecular subtypes of HNSCC based on hypoxia-related genes. The relationships between molecular subtypes and prognosis and clinical features were further evaluated. Furthermore, we generated a prognostic risk model with differentially expressed genes (DEGs) between the distinct hypoxia-immune subtypes. The risk model showed good predictive performance for the prognosis and diagnosis of patients with HNSCC.

## Results

### Establishment of hypoxia-related molecular subtypes in HNSCC

**Figure 1:**
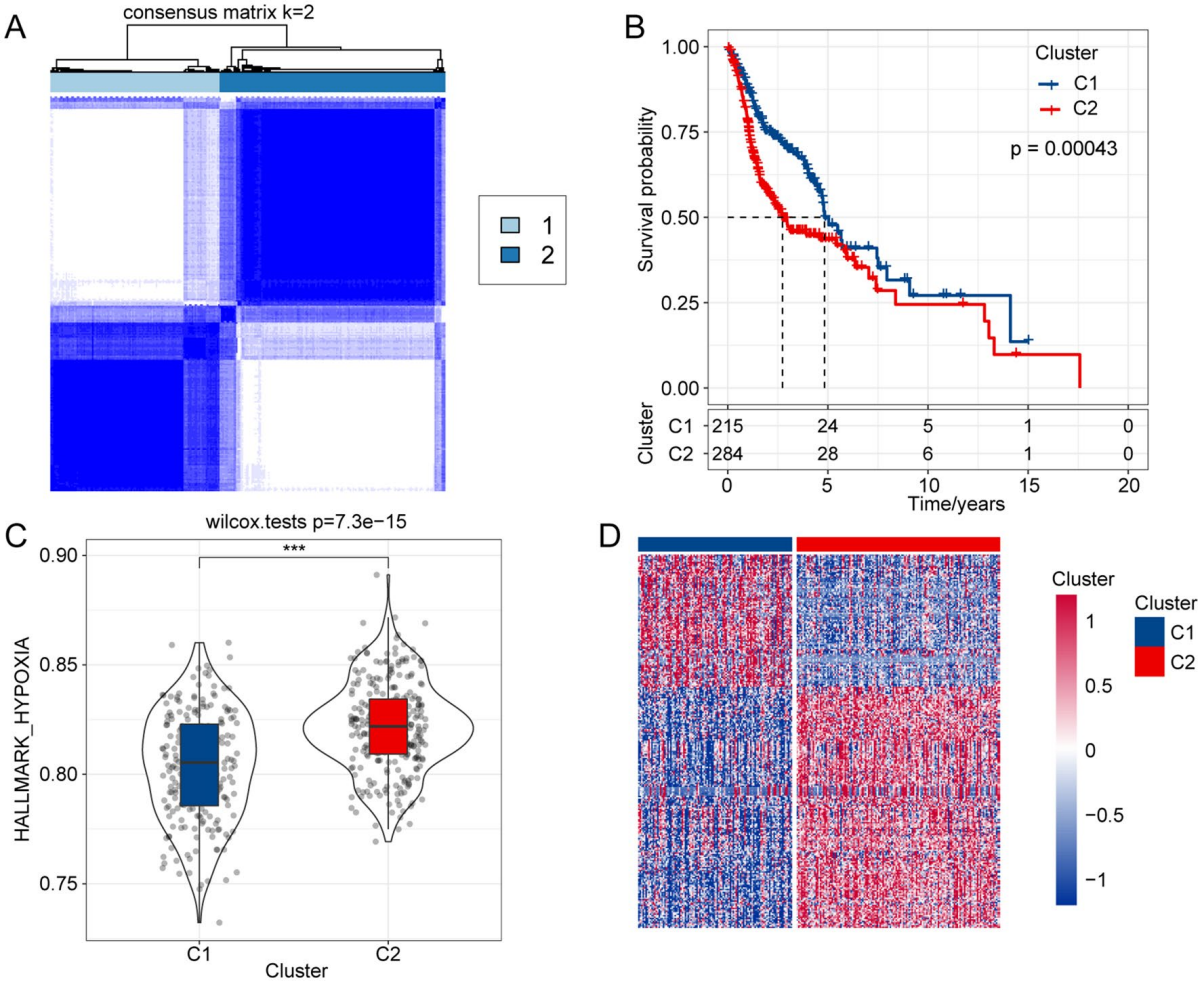
Identification of hypoxia-related molecular subtypes in HNSCC. (**A**) Two hypoxia-related subtypes were identified by ConsensusClusterPlus clustering analysis. (**B**) Survival curve analysis of the two distinct molecular subtypes. (**C**) ssGSEA of the molecular subtypes. (**D**) Analysis of differentially expressed genes between molecular subtypes by the limma R package.

We designed and conducted our study as shown in the flow chart in Supplementary Fig. 1. First, we collected the mRNA data of 200 hypoxia-related genes. Second, a total of 61 genes associated with HNSCC prognosis were screened using univariate Cox regression analysis. Next, we divided the HNSCC patients in the TCGA into different subtypes based on the expression of the 61 prognosis-related genes using the ConsensusClusterPlus R package, and two distinct subtypes were identified: cluster 1 (C1) and cluster 2 (C2) (Fig. 1A). In addition, prognostic analysis of the two subtypes demonstrated that patients in C1 had a better prognosis than those in C2 (Fig. 1B). Additionally, to explore the association between biological function and hypoxia-related subtypes, we performed GSVA enrichment analysis to calculate the HALLMARK-HYPOXIA score of each sample. As shown in Fig. 1C, the HALLMARK-HYPOXIA score in C2 was significantly higher than that in C1. We then used the limma R package to identify the DEGs between C1 and C2, and we found that 287 of 446 DEGs were upregulated in C2 (false discovery rate (FDR) < 0.05, |log2 fold change (FC)|> 1, Fig. 1D).

### Identification of subgroups of hypoxic immune microenvironments

**Figure 2:**
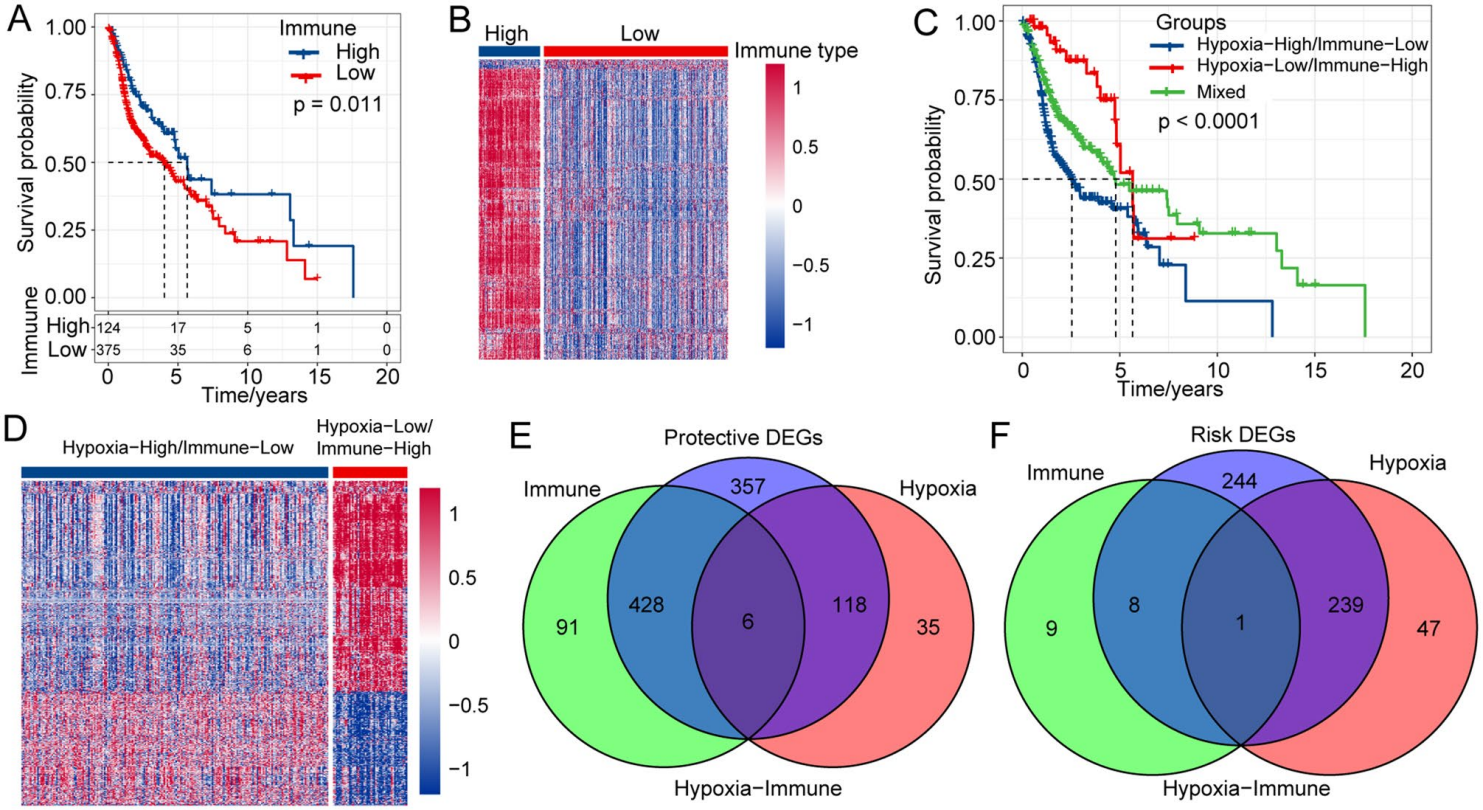
Identification of subgroups of hypoxic immune microenvironments. (**A**) Survival analysis of the immunization group. (**B**) Analysis of differentially expressed genes in the immunization group. (**C**) Survival curve of hypoxic immune microenvironment grouping. (**D**) Heatmap of differentially expressed genes among hypoxic immune microenvironmental groups, and R software v3.5.0 (version 3.6.1) was adopted to drawn the heat map. (**E**) Identification of protective differential genes. (**F**) Identification of risk differential genes.

Ample evidence has demonstrated that hypoxia plays an essential role in regulating immune cell function^14,15^. Here, we used the ESTIMATE R package to calculate the immune infiltration score of each patient in the TCGA database, and the survminer package was used to find the best segmentation point. Then, the patients were classified into two distinct immune subgroups (immune-high group and immune-low group). Next, we performed Kaplan–Meier survival analysis to calculate the survival curves of the immune groups. The results showed that the overall survival time of patients in the immune-high subgroup was significantly longer than that of patients in the immune-low subgroup (Fig. 2A). We analyzed the DEGs between the immune-high and immune-low subgroups using the limma package (FDR < 0.05, |log2FC|> 1). A total of 543 DEGs were screened, among which 525 genes were upregulated and 18 genes were downregulated in the immune-high subgroup (Fig. 2B). A total of 499 patients with HNSC were used in the TCGA dataset in our study. In details, 72 patients with HPV-positive, 30 patients with HPV-negative, and 397 patients with unknown HPV types were adopted based on TCGA-HNSC cohort. The percent of HPV-positive, HPV-negative and unknown HPV status in three subtypes was showed in Supplementary Fig. 2. Through the analysis above, we found that patients in C2 with high HALLMARK-HYPOXIA scores have a poor prognosis, and those in the immune-low group with low immune infiltration scores have a poor prognosis, suggesting that HNSCC patients with both low HALLMARK-HYPOXIA scores and high immune infiltration scores have worse prognosis. Therefore, we classified the patients into the hypoxia-high/immune-low subgroup and the hypoxia-low/immune-high subgroup and placed other samples in the mixed subgroup. As expected, among them, patients in the hypoxia-high/immune-low subgroup had the worst prognosis, while those in the hypoxia-low/immune-high subgroup had the best prognosis (Fig. 2C). Furthermore, we used the limma package to analyze the DEGs between the hypoxia-high/immune-low and hypoxia-low/immune-high subgroups. A total of 492 genes were upregulated in the hypoxia-high/immune-low subgroup, and 909 genes were downregulated in the hypoxia-low/immune-high subgroup (Fig. 2D). Finally, we intersected the downregulated DEGs between the hypoxia-high/immune-low and hypoxia-low/immune-high subgroups with the downregulated genes of the molecular subtypes and the upregulated genes in the immune subtypes to obtain 434 protective DEGs (Fig. 2E). We intersected upregulated DEGs between the hypoxia-high/immune-low and hypoxia-low/immune-high subgroups with the upregulated genes of molecular subtypes and downregulated immune genes to obtain 248 risk DEGs (Fig. 2F).

### Functional annotation of protective DEGs and risk DEGs

**Figure 3:**
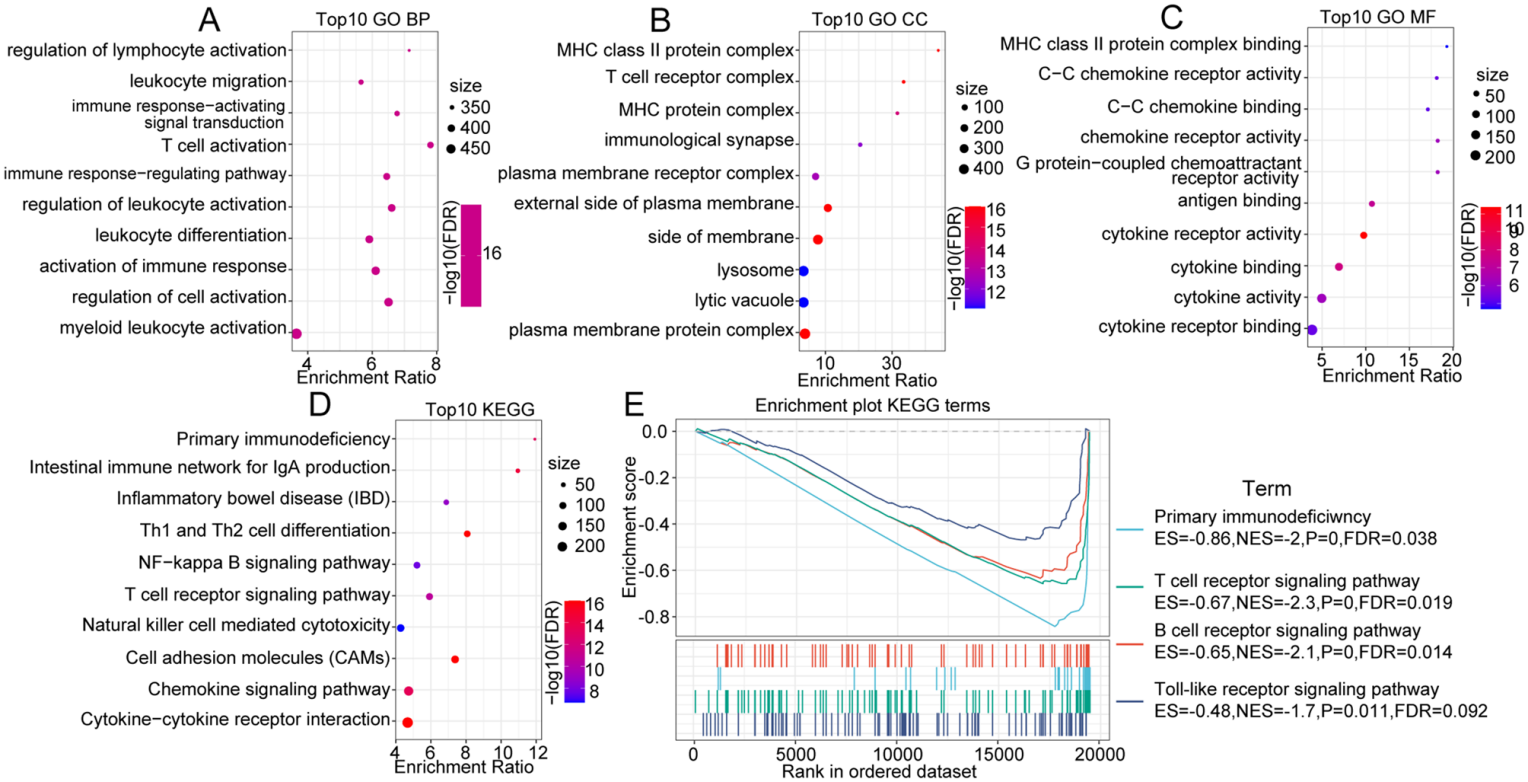
(**A**) BP annotation map of protective DEGs. (**B**) CC annotation map of protective DEGs, and R software v3.5.0 (version 3.6.1) was adopted to drawn the annotation map. (**C**) MF annotation map of protective DEGs. (**D**) KEGG annotation map of protective DEGs, and R software v3.5.0 (version 3.6.1) was adopted to drawn the annotation map. (**E**) GSEA of pathways enriched in hypoxic immune grouping. Abbreviations: BP, biological process; CC, cellular component; MF, molecular function; KEGG, Kyoto Encyclopedia of Genes and Genomes.

To better characterize the protective DEGs and risk DEGs, KEGG and GO functional enrichment analyses were performed. For the GO functional annotations of 434 protective DEG genes, 610 functional annotations of BPs were found to have significant differences, and the results of the top 10 annotations are shown in Fig. 3A. Approximately 60 CC terms with significant differences were annotated, and the top 10 items are shown in Fig. 3B. A total of 55 MF terms with significant differences were annotated (FDR < 0.05); the annotation results of the top 10 terms are shown in Fig. 3C. Additionally, for the KEGG pathway enrichment of 434 protective DEGs, approximately 41 pathways were significant (FDR < 0.05). The top 10 annotations are shown in Fig. 3D. For the GO functional annotations of 248 risk DEGs, the top 10 BP, MF and CC annotations with significant differences are shown in Supplementary Fig. 3A–C. For the enrichment of the KEGG pathways of 248 risk DEGs, in the top 10 annotations, we found several significantly different tumor-related pathways, such as focal adhesion, ECM-receptor interaction, small cell lung cancer, and the PI3K-Akt signaling pathway (Supplementary Fig. 3D). The results clearly showed that HNSCC patients could be divided into distinct subtypes through complex pathways.

In the TCGA-HNSC dataset, we also used GSEA to analyze the significantly enriched pathways in the hypoxia-high/immune-low and hypoxia-low/immune-high subgroups, and we selected the gene set c2.cp.kegg.v7.0. symbols.gmt, which contains KEGG terms. The thresholds for selecting enriched pathways were *p* < 0.05 and FDR < 0.25. The results showed that in the hypoxia-low/immune-high subgroup, immune-related pathways were more enriched, such as primary-immunodeficiency, T cell receptor signaling, and toll like receptor signaling pathway (Fig. 3E), suggesting that the hypoxia-low/immune-high subgroup was more correlated with immunity than the hypoxia-high/immune-low subgroup.

### Association between hypoxia-immune subgroups and the tumor immune microenvironment

**Figure 4:**
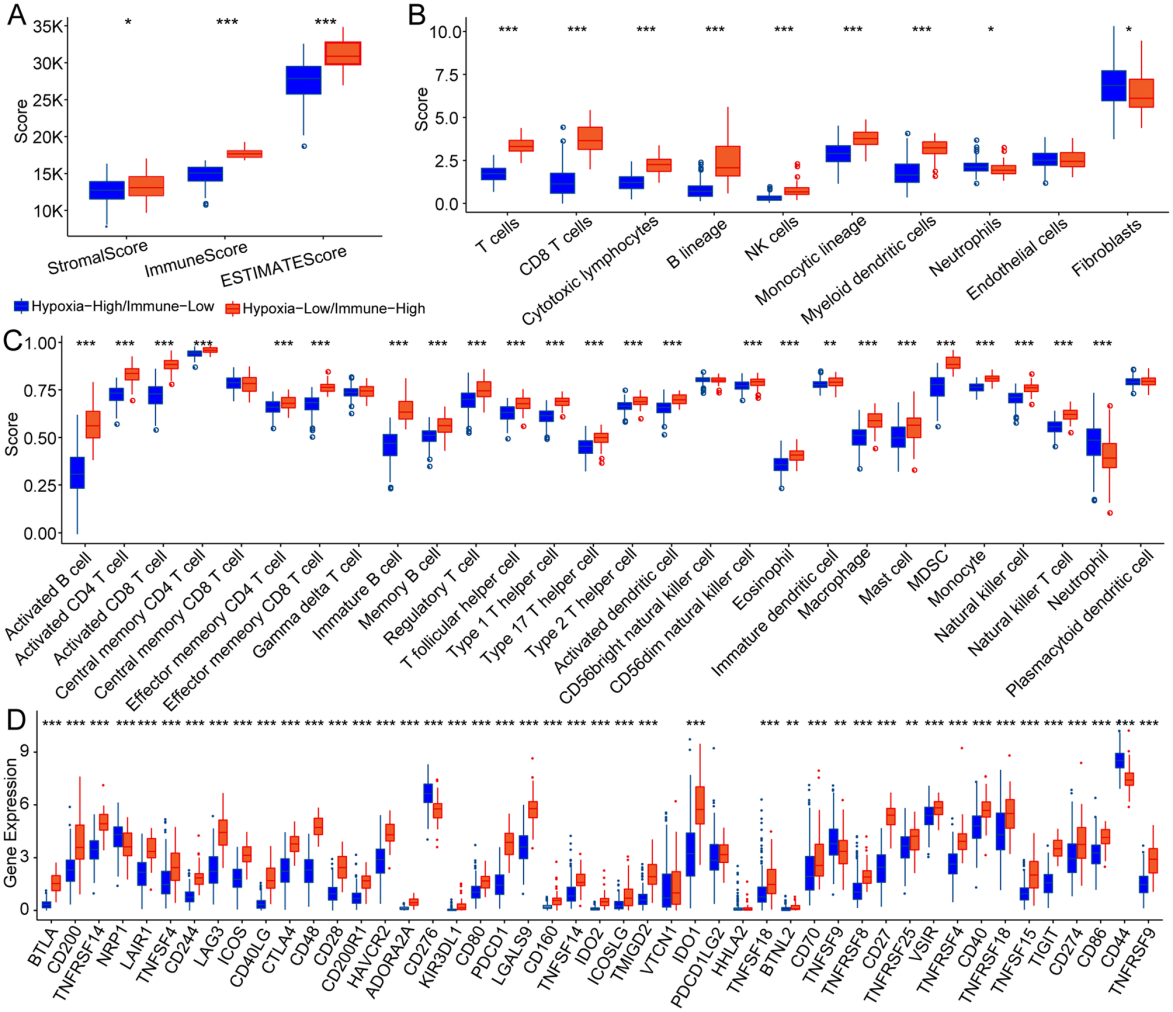
Immune microenvironment analysis and immune checkpoint analysis of hypoxia-immune groups. (**A**) Comparison of differences in immunological scores between different hypoxia-immune groups. (**B**) Comparison of differences in the MCP-counter immunity score between hypoxia-immune groups. (**C**) Comparison of ssGSEA immune score differences between hypoxia-immune groups. (**D**) Comparison of differences in immune checkpoints between hypoxia-immune groups. *p < 0.05, **p < 0.01, ***p < 0.001.

To investigate the effect of immunity and hypoxia on the tumor immune microenvironment, we evaluated the immune scores of each patient and then compared their differences between the hypoxia-high/immune-low and hypoxia-low/immune-high groups using the ESTIMATE, MCP-counter, and ssGSEA methods. Consistent with the previous results (Fig. 2A), the immune scores in the hypoxia-low/immune-high group were higher (Fig. 4A–C). In addition, we collected 47 immune checkpoints and compared the differences in these immune checkpoints between these groups. The results showed that 44 (93.62%) of these genes were different, most of which were upregulated in the hypoxia-low/immune-high group, including CD276, CD70, LAG3, CTLA4, PDCD1, CD86, and IDO1 (Fig. 4D).

### Differential analysis of immunotherapy and chemotherapy for hypoxic immune groups

**Figure 5:**
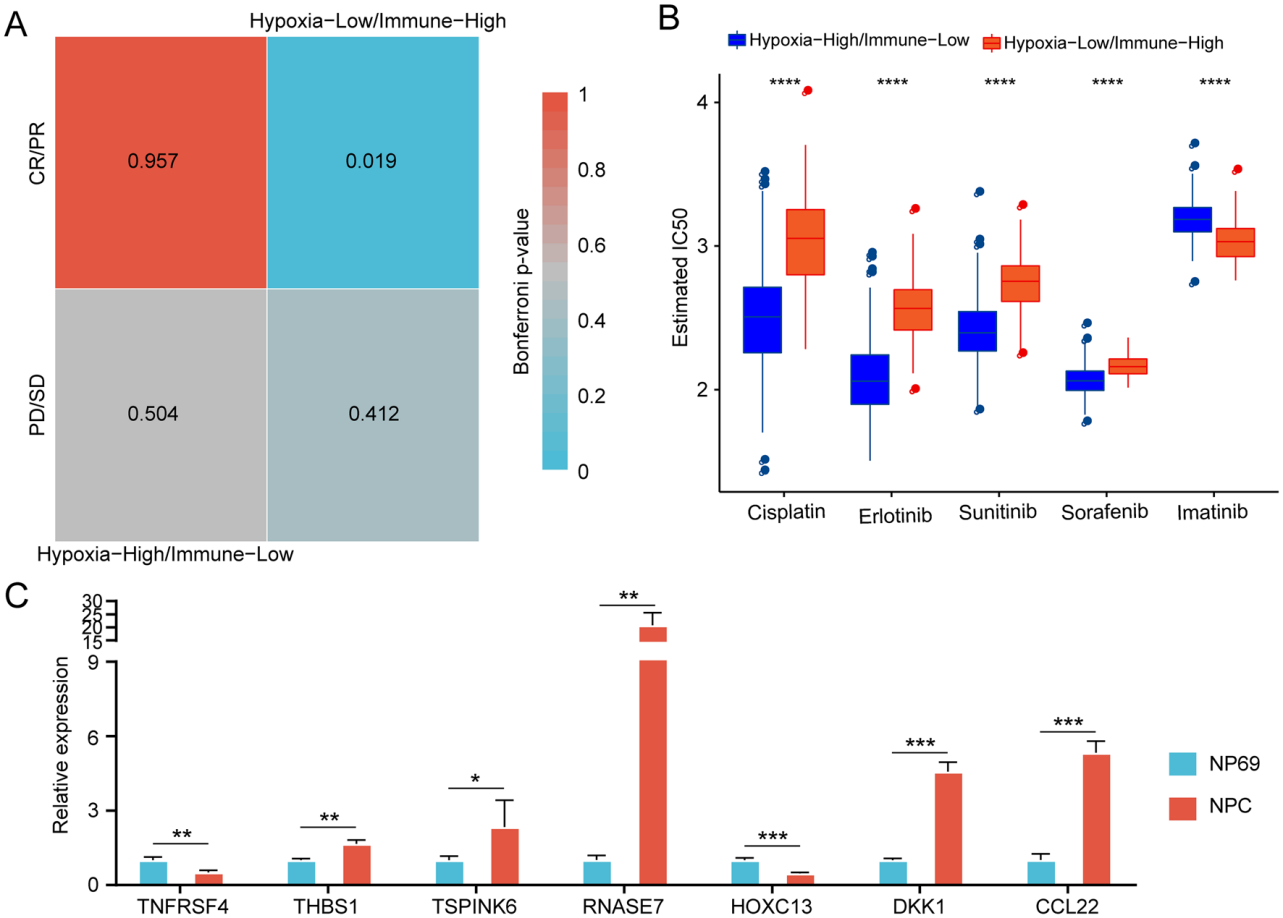
Differential analysis of immunotherapy and chemotherapy for hypoxic-immune subtypes. (**A**) Submap analysis showed that IC1 could be more sensitive to anti-PD-L1 treatment. (**B**) Box plots of the estimated IC50 values for cisplatin, erlotinib, sunitinib, sorafenib, and imatinib. (**C**) qRT-PCR analysis showed the expression levels of the key genes in NPC and NP69 cell lines. *p < 0.05, **p < 0.01, ***p < 0.001.

Next, we sought to further analyze the differences in immunotherapy and chemotherapy between different hypoxic immune groups. Here, we used the subclass mapping method to compare the similarity between the subtypes in the IMvigor210 dataset to investigate the clinical activity of PD-L1 blockade. The lower the p value was, the higher the similarity. As a result, we found that patients in the hypoxia-low/immune-high group were more sensitive to anti-PD-L1 treatment (Bonferroni-corrected *p* < 0.05, Fig. 5A). In addition, we analyzed the response of patients with different subtypes to traditional chemotherapy drugs, such as cisplatin, erlotinib, sunitinib, sorafenib, and imatinib. We found that patients in the hypoxia-low/immune-high group were more sensitive to cisplatin, erlotinib, sunitinib, and sorafenib (Fig. 5B). All the results indicated that hypoxic immune groups might contribute to providing guidance for immunotherapy and chemotherapy in patients with HNSCC.

### Construction of a prognostic risk model based on DEGs between hypoxia-immune subtypes

Based on the 782 protective and risk DEGs, we performed univariate Cox regression analysis and found that 98 DEGs were associated with prognosis. To further compress these 98 genes, we used LASSO regression analysis. We finally identified 7 genes, including THBS1, RNASE7, DKK1, SPINK6, HOXC13, TNFRSF4 and CCL22.

The final 7-gene signature formula was as follows:

$$\begin{gathered} {\text{Risk score}} = 0.125*{\text{THBS1}} + 0.151*{\text{RNASE7}} + 0.076*{\text{DKK1}} - 0.187*{\text{SPINK6}} \hfill \\ \quad \quad \quad \quad \quad + 0.206*{\text{HOXC13}} - 0.213*{\text{TNFRSF4}} - 0.128*{\text{CCL22}} \hfill \\ \end{gathered}$$

**Figure 6:**
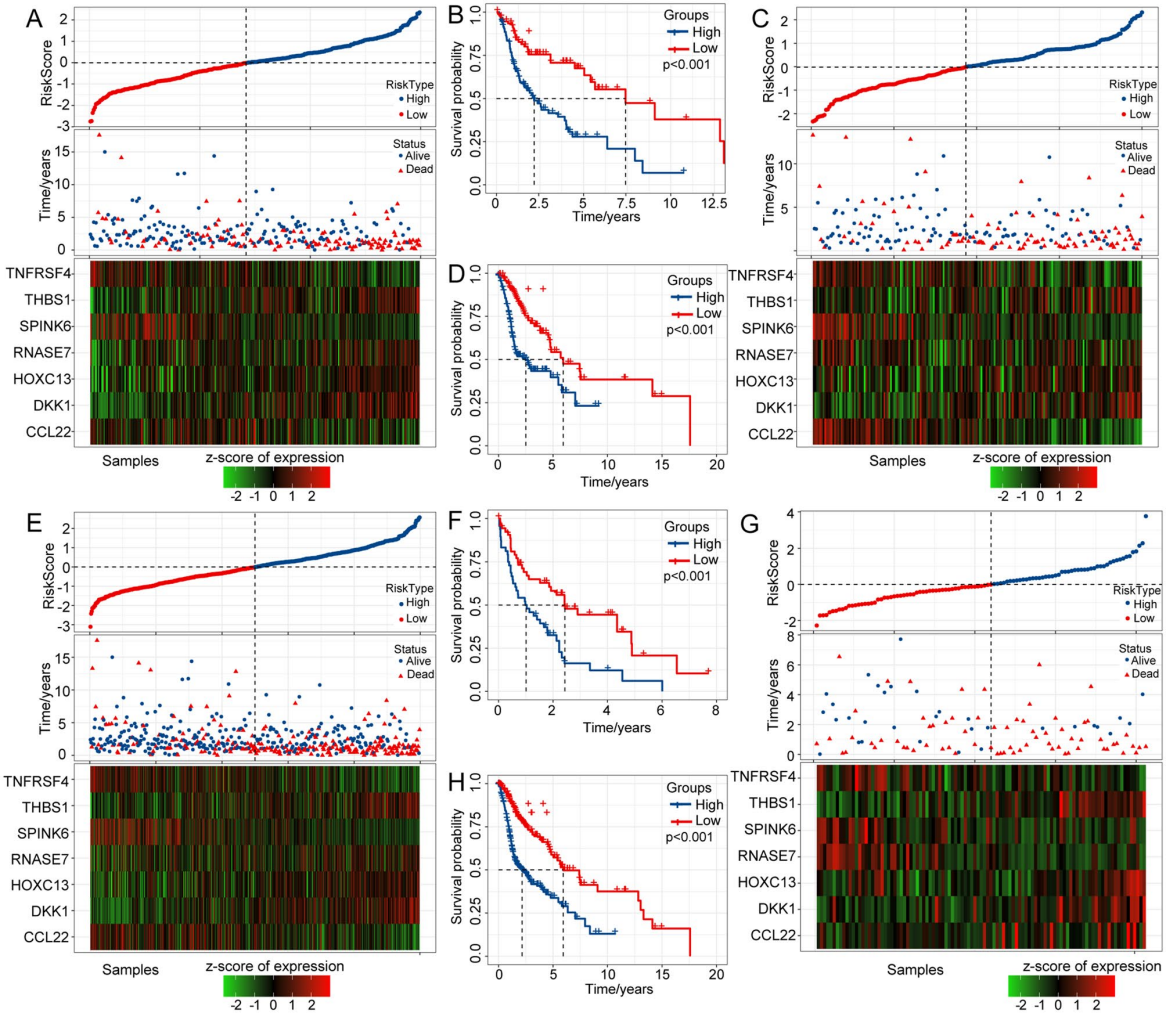
Construction and validation of a prognostic risk model based on DEGs between hypoxia-immune subtypes.

First, we explored the expression level of the 7 gene in cell lines. The expression level of the 7 genes in human immortalized nasopharyngeal epithelial cell NP69 and Human nasopharyngeal carcinoma cell NPC was detected by qRT-PCR analysis. Compared with NP69 cell, 5 of 7 genes including THBS1, SPINK6, RNASE7, DKK1, and CCL22 were upregulated in NPC cell. (Fig. 5C) Then, we calculated the risk score of each patient in the TCGA training dataset and plotted the distribution of the risk scores. The results indicated that patients with a high risk score had a poor prognosis (Fig. 6A). Furthermore, survival analysis showed that patients in the low-risk group had a better prognosis than patients in the high-risk group (*p* < 0.0001, Fig. 6B). The results indicated the good performance of the established 7-gene signature for predicting HNSCC prognosis. To validate the stability of the 7-gene signature, a similar workflow was employed for the validation set, wherein three datasets (TCGA validation set, whole TCGA dataset, and GSE42743 dataset) were analyzed. The distribution of risk scores and Kaplan–Meier curves of the 7-gene signature for the TCGA validation set (Fig. 6C, D), whole TCGA dataset (Fig. 6E, F), and GSE42743 dataset (Fig. 6G, H) further validated that this risk model could accurately predict the prognosis of HNSCC patients.

### Excellent prognostic efficacy of the 7-gene signature for patients with different clinical features

**Figure 7:**
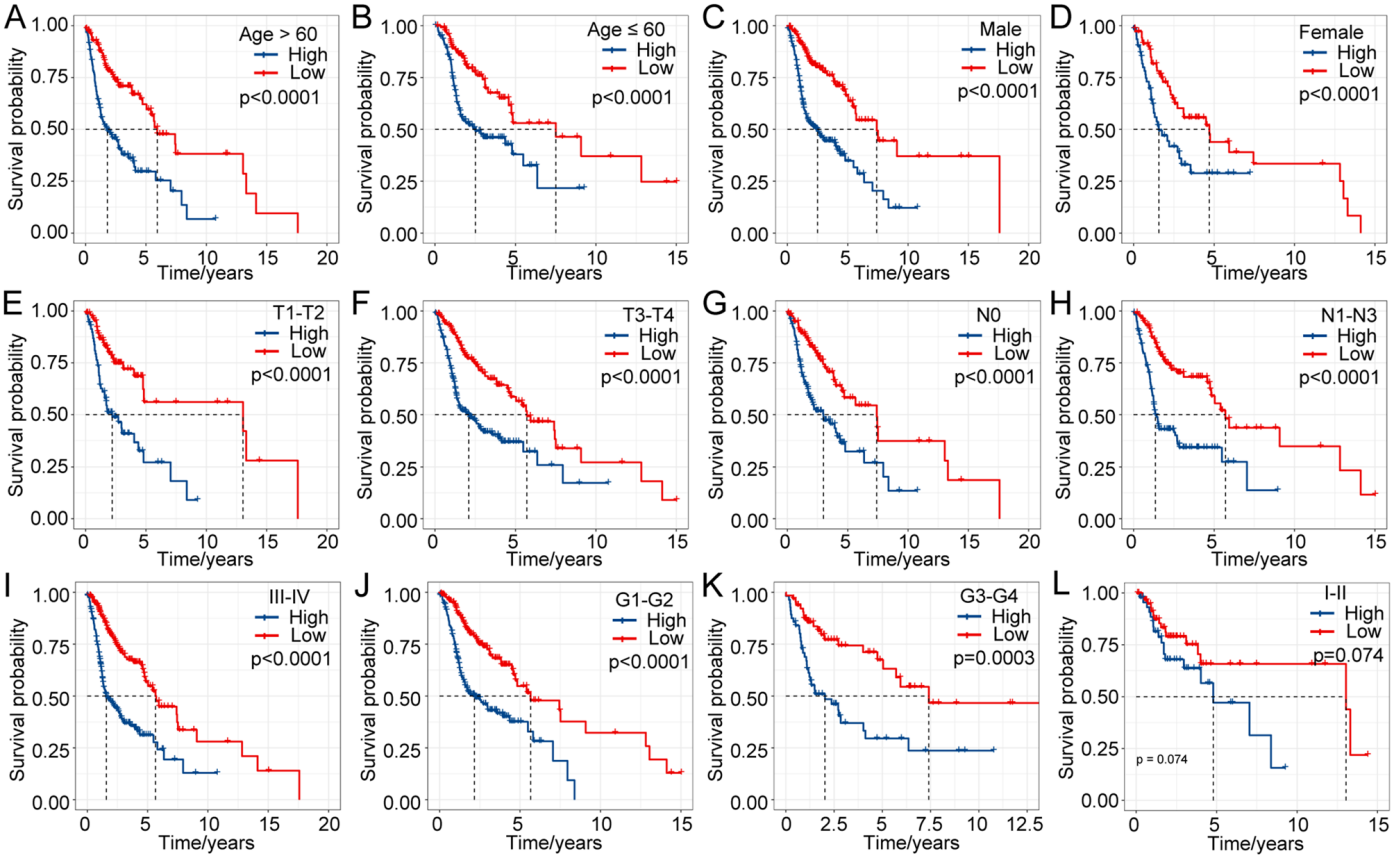
Prognostic efficiency of the risk model in patients with different clinical characteristics in the TCGA dataset.

Furthermore, the associations of the risk score with different clinical characteristics of the TCGA cohort were analyzed. The seven-gene signature discriminated high-risk patients with poor prognosis in different subgroups with different clinical characteristics (age, sex, TNM stage, grade and p stage), and the results are shown in Fig. 7A–L. We compared the distribution of the risk score among the clinical characteristics of the TCGA dataset and found that there were no significant differences in age, sex, N stage and M stage (*p* > 0.05, Supplementary Fig. 4A–D), while there were significant differences in other characteristics, including T stage, stage, and grade (*p* < 0.05, Supplementary Fig. 4E–G). All the results further validate that the 7-gene signature has excellent predictive ability for HNSCC patients with different clinicopathological features.

### The 7-gene signature is an independent risk factor for patients with HNSCC

**Figure 8:**
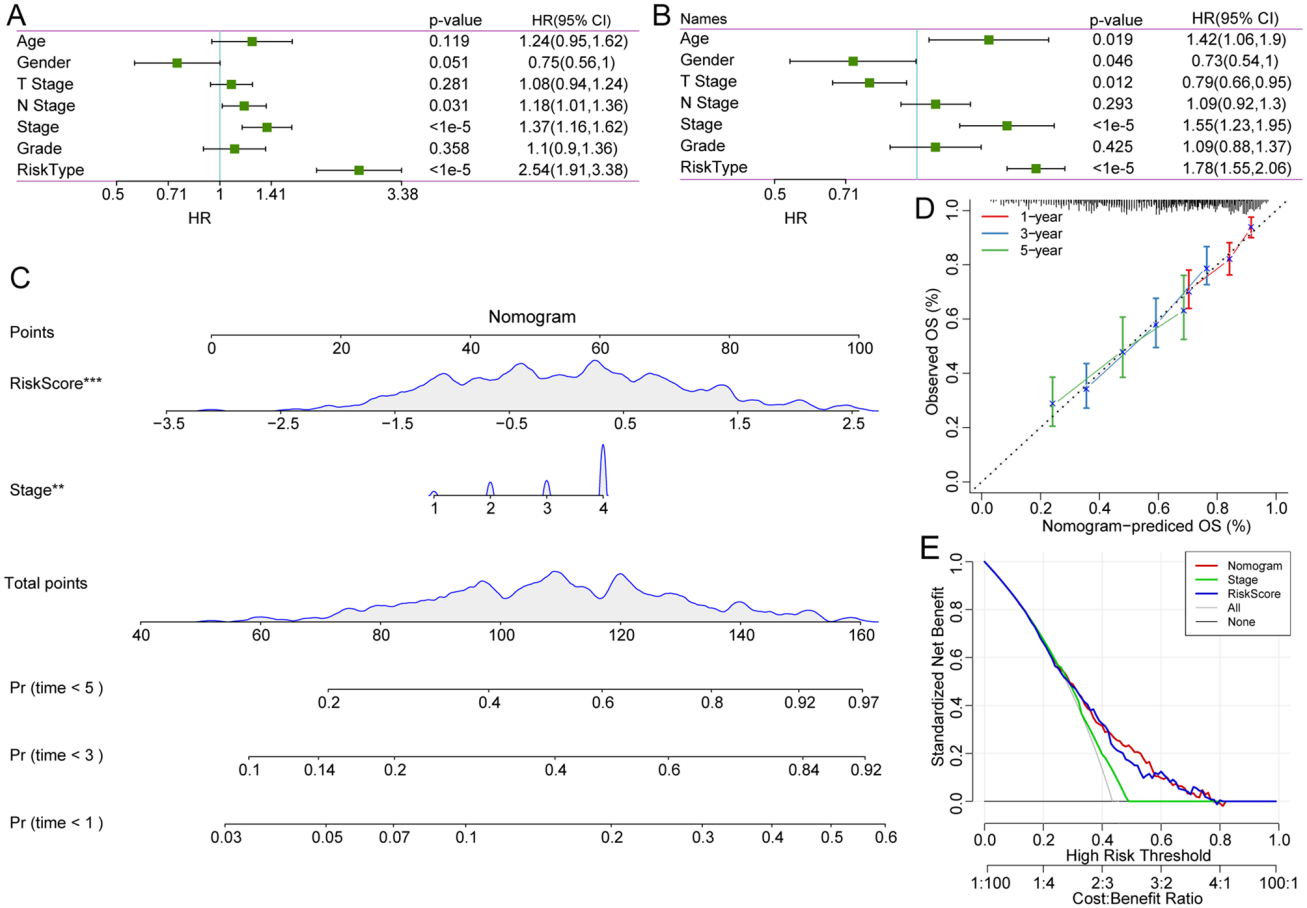
Construction of the nomogram for predicting the prognosis of HNSCC patients in the TCGA-HNSC cohort. Univariate Cox regression analysis (**A**) and multivariate Cox regression analysis (**B**) showed that the seven-gene signature was an independent risk factor. (**C**) Nomogram constructed by the risk score and stage in HNSCC patients. (**D**) Survival rate correction curves of the nomogram. (**E**) DCA curves of the nomogram, stage, and risk score showed that the nomogram had good predictive performance.

To explore the prognostic efficacy of the 7-gene signature model for HNSCC patients, we analyzed clinical data, including age, sex, T stage, N stage, stage and risk score. In the TCGA dataset, univariate Cox regression analysis revealed that the risk score was significantly related to survival (Fig. 8A), and multivariate Cox regression analysis further demonstrated that the risk score was still significantly associated with survival time (HR = 1.78, 95% CI = 1.55–2.06, *p* < 1e-5, Fig. 8B).

Nomograms are an intuitive and effective way to display the results of risk models, and they are especially convenient to apply in predicting the survival outcome. In a nomogram, the length of the straight line indicates the degree of influence of different variables on the outcome and the influence of different values of the variables on the outcome. We built a nomogram model integrating stage and the risk score using the TCGA dataset and found that the risk score had a great influence on the survival rate prediction (Fig. 8C). Additionally, we corrected the nomogram data for 1, 3, and 5 years to visualize the performance of the nomogram (Fig. 8D). Decision curve analysis (DCA) curves of the nomogram, stage, and risk score indicated that our model has good predictive performance for the prognosis of patients with HNSCC (Fig. 8E).

## Discussion

Hypoxia is a typical characteristic of the TME^16,17^. In recent years, many groups have focused on the TME, in which immune cells play a crucial role in the progression of cancer^18,19^. In this study, we collected the mRNA data of 200 hypoxia-regulated genes and downloaded the mRNA expression profiles and clinical information of patients with HNSCC from the TCGA and GEO databases. We identified two distinct molecular subtypes, C1 and C2, through the ConsensusClusterPlus method. In addition, we used ESTIMATE analysis to calculate the immune score of each patient in the TCGA cohort and defined three distinct hypoxia-immune subgroups (hypoxia-high/immune-low, hypoxia-low/immune-high and mixed subgroups). Further gene enrichment analysis of the genes in these subgroups showed that immune-related pathways were enriched in the hypoxia-low/immune-high group. In another study, Khouzam et al. generated an eight-gene hypoxia signature based on the published literature and found that the high hypoxia state of pancreatic ductal adenocarcinoma indicates an immunosuppressed TME^20,21^ used consensus clustering analysis to build a risk model based on hypoxia-related genes and found that these genes were closely correlated with the infiltration of various immune cell types. All this evidence for the association between hypoxia and immunity might contribute to predicting prognosis and guiding treatment for patients with cancers.

In the present study, we identified seven DEGs (THBS1, RNASE, DKK1, HOXC13 CCL2, TNFRSF4 and SPINK6) associated with hypoxia in HNSCC patients that may serve as potential biomarkers of HNSCC prognosis. Based on these 7 DEGs, we generated a risk model and found that CCL2, TNFRSF4 and SPINK6 were negatively correlated with the risk score, while THBS1, RNASE, DKK1, and HOXC13 were positively correlated with the risk score. Our results further show that patients with high risk scores have a poor prognosis compared with those with low scores. Univariate and multivariate Cox regression analyses showed that the risk model is an independent prognostic biomarker for patients with HNSCC.

Previous studies have reported that thrombospondin-1 (THBS1) is not a tumor suppressor gene, but the expression of THBS1 in tumor cells is regulated by oncogenes and tumor suppressor genes^22^. Burkitt et al. reported that the regulation of TSP1 by hypoxia varies with the TME. TSP1 was highly expressed in colorectal cancer cells when HIFα was lacking, which inhibited protumor angiogenesis^23^. Dickkopf-related protein 1 (DKK1) is an inhibitor of the Wnt/β-catenin signaling pathway and was previously considered a tumor suppressor. However, recent studies have shown that upregulated DKK1 is correlated with progression^24^. We showed for the first time that RNASE, HOXC13, CCL2, TNFRSF4 and SPINK6 were correlated with HCC outcomes. A better understanding of the biological functions of the 7 genes in HNSCC is needed for the further development of novel biomarkers for the prognosis of HNSCC.

## Conclusions

We identified new hypoxia-immune HNSCC subtypes, among which patients in the hypoxia-low/immune-high subgroup could be more sensitive to immunotherapy and chemotherapy than other subgroups. In addition, we constructed a prognostic model based on hypoxia-related genes that has high prognostic prediction accuracy in patients with HNSCC.

## Materials and methods

### Data preparation and data processing

We downloaded the mRNA expression profiles and clinical data of patients with HNSCC from the TCGA and GEO databases. Patients with incomplete information were excluded. There were 499 TCGA-HNSC samples, 270 GSE65858 samples, and 103 GSE42743 samples. Detailed clinical information is shown in Supplementary Table 1. We randomly divided the 499 samples of the TCGA dataset into a training dataset and a validation dataset in a 3:2 ratio. The final training dataset included 299 samples, and the training dataset included 200 samples. The training dataset and validation dataset sample information are shown in Supplementary Table 2.

We downloaded the genes of hypoxia-related pathways (HALLMARK_HYPOXIA) from the Molecular Signatures Database (MSigDB) v7.0 and identified a total of 200 genes related to hypoxia.

The immunotherapy dataset IMvigor210 includes tumor samples that were subjected to PD-L1 testing^25^. Here, we analyzed the differences in immunotherapy responses of different groups based on the IMvigor210 dataset.

### Culturing of cells and reverse transcription‑quantitative polymerase chain reaction (RT‑qPCR)

Human immortalized nasopharyngeal epithelial cell NP69 and Human nasopharyngeal carcinoma cell NPC were obtained from Shanghai FuHeng Biotechnology Co., Ltd. (Shanghai, China). Cell NPC was cultured with DMEM with 10% FBS (Gibco, Rockville, MD, USA). The cell line NP69 was cultured in KM medium. All cell lines were grown at 37°Cwith 5% CO2.

Total RNA was isolated from cell NP69 and NPC using Trizol reagent (Invitrogen). cDNA was synthesized using PrimeScript RT Reagent Kit (TaKaRa, Tokyo, Japan). The mRNA expression levels were detected by qRT-PCR using the SYBR-Green (Takara). Relative gene expression levels were analyzed using the 2 − ΔΔCt method. Gene-specific primers were showed in Supplementary Table 3.

### Gene set variation analysis (GSVA)

GSVA (http://www.bioconductor.org) is a nonparametric, unsupervised algorithm that was conducted to assess the enrichment score of a specific gene set in each sample^26^. GSVA transforms gene expression data from a single gene as a characteristic expression matrix to a specific gene set as a characteristic expression matrix, which makes subsequent statistical analysis more convenient. In this study, we used the GSVA R package to perform single-sample gene set enrichment analysis (ssGSEA) based on the gene expression profile of each sample in the TCGA training dataset. Then, we obtained the score of each sample for the different functions of the HALLMARK data.

### Kyoto encyclopedia of genes and genomes (KEGG) and gene ontology (GO)

KEGG (https://www.genome.jp/kegg/kegg.html) is a knowledge base of genes and genomes that is used to assign functional meanings to genes and genomes^27,28^ GO is an ontology widely used in the field of bioinformatics that covers three aspects of biology, including biological process (BP), cellular component (CP), and molecular function (MF). To explore the potential functions of the DEGs, we used KEGG (https://www.kegg.jp/) and GO (http://geneontology.org/) to analyze the protective DEGs and risk DEGs. The WebGestaltR (v0.4.2) R package was used to analyze and annotate the genes. *P* < 0.05 was considered statistically significant.

### Estimation of STromal and immune cells in MAlignant tumor tissues using expression data (ESTIMATE)

ESTIMATE is an algorithm that uses expression profile data to calculate stromal cell and immune cell scores to predict the abundance of these two types of cells in tumors. Here, we used the ESTIMATE R package to calculate the immune score for each patient in the TCGA-HNSC dataset. The Survminer package was used to identify the optimal segmentation point, which was used to divide patients into high- and low-immunity groups. In addition, we used ESTIMATE to compare the immune score of each patient in different subgroups.

### Microenvironment cell population–counter (MCP-counter)

MCP-counter, which was introduced by the Becht team in 2016, allows the robust quantification of the absolute abundance of immune cells and stromal cells in tumor tissues from transcriptomic data^29^. It is widely used to draw a global picture of immune cell infiltration across tumor tissues and normal control tissues. In this study, we used MCP-counter to calculate the immune score of each patient in distinct subgroups.

### Least absolute shrinkage and selection operator (LASSO)

LASSO is a popular method of compressed estimation^30,31^. Previous studies have reported that LASSO Cox regression analysis is a useful method to construct gene-based models^32,33^. Here, with the glmnet R package, we used the LASSO algorithm to compress the number of genes in the gene signature model.

### Statistical analysis

All data analyses were performed with GraphPad Prism v7.0 (GraphPad, San Diego, CA, USA) or R software v3.5.0 (version 3.6.1). And R software v3.5.0 (version 3.6.1) was adopted to drawn the heat maps of the figures. Univariate and multivariate Cox regression analyses were used to screen independent prognostic factors. The Kaplan–Meier method and log-rank test were employed for survival analysis. The prognostic efficacy of the variables was evaluated using time-dependent receiver operating characteristic (ROC) curve analysis. Differences in two groups were analyzed by Student’s t-test, and comparisons of three or more groups were analyzed using one-way ANOVA.

## Supplementary Information

Supplementary Information 1.

Supplementary Information 2.

Supplementary Information 3.

Supplementary Information 4.

Supplementary Information 5.

Supplementary Information 6.

Supplementary Information 7.

Supplementary Information 8.

## Data Availability

The datasets used and/or analysed during the current study available from the corresponding author on request.
