# Supplementary figures and images for: Construction of a hypoxia-derived gene model to predict the prognosis and therapeutic response of head and neck squamous cell carcinoma
Source: Sci Rep. 2022 Aug 8;12:13538. doi: 10.1038/s41598-022-17898-2 (PMC9363468; doi:10.1038/s41598-022-17898-2)

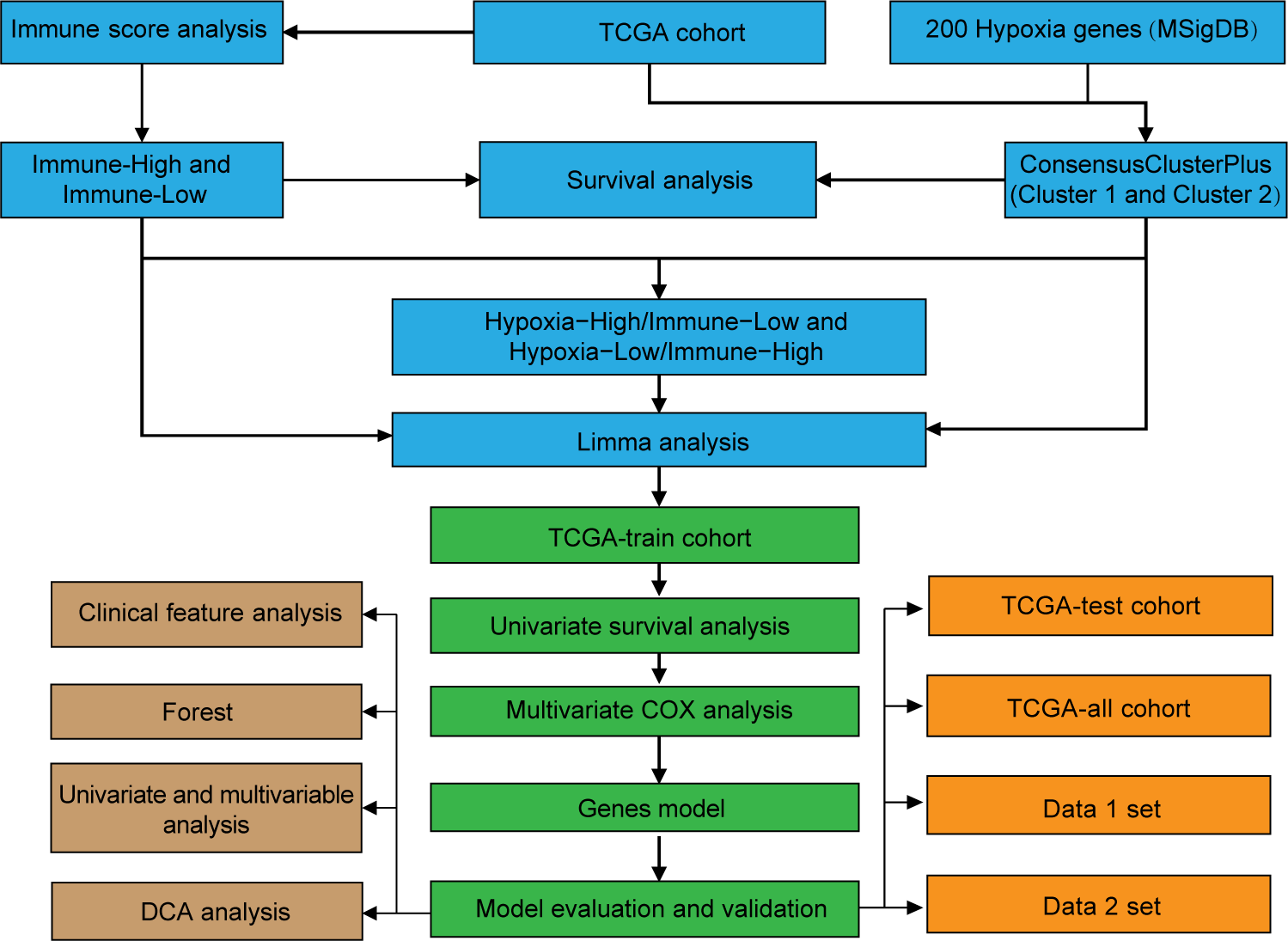

Supplement: Supplementary file 1 — Supplementary Information 1. [file 41598_2022_17898_MOESM1_ESM.tif]

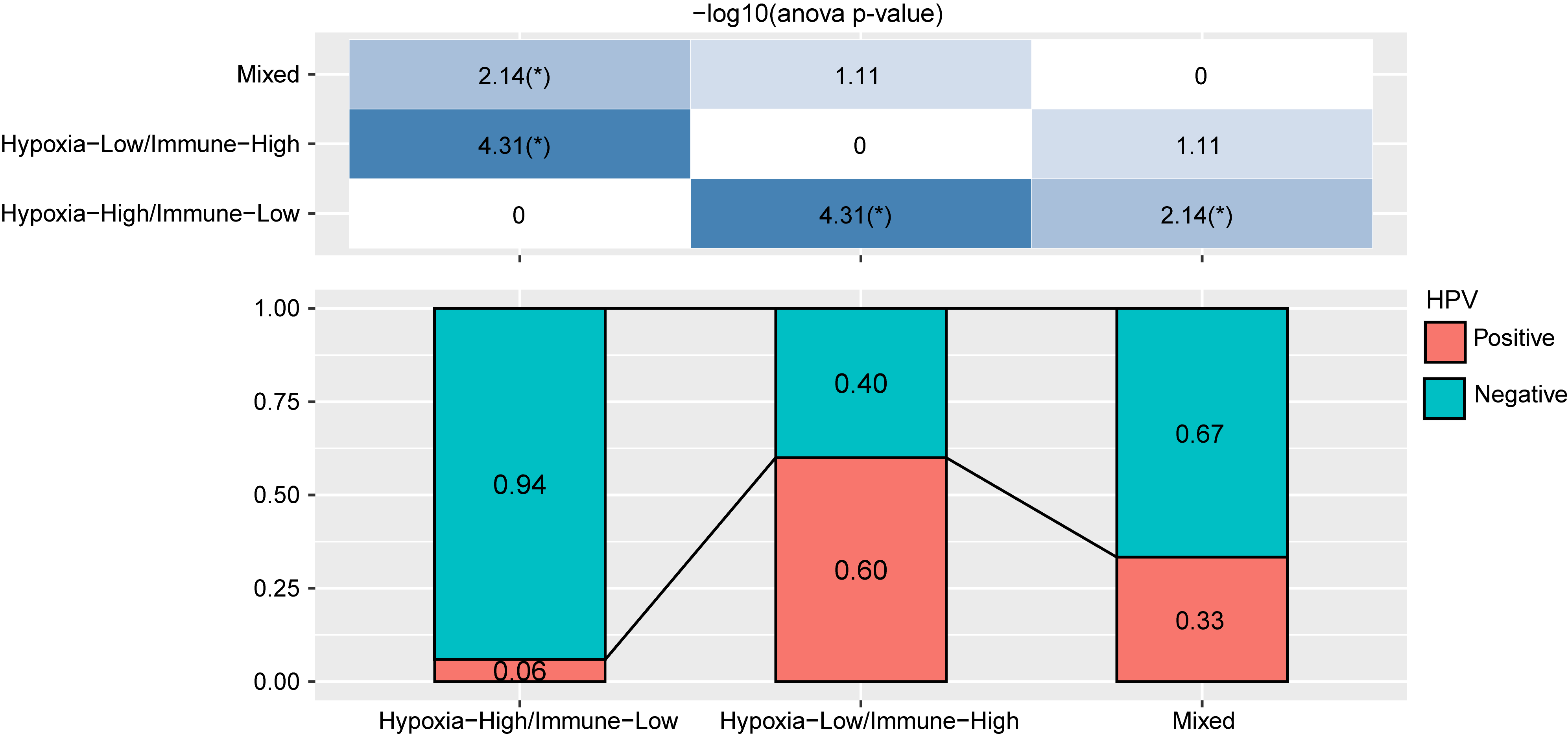

Supplement: Supplementary file 2 — Supplementary Information 2. [file 41598_2022_17898_MOESM2_ESM.tif]

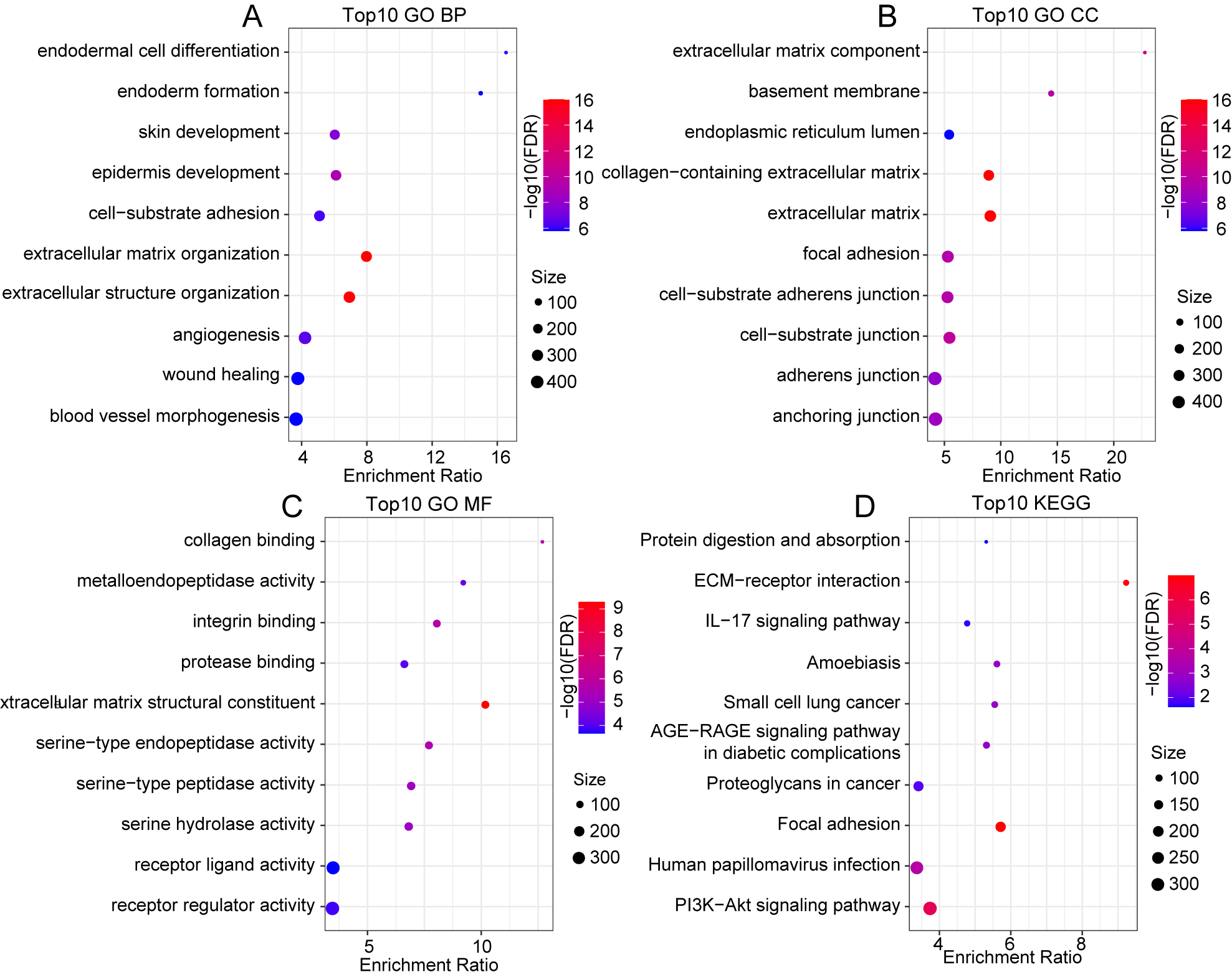

Supplement: Supplementary file 3 — Supplementary Information 3. [file 41598_2022_17898_MOESM3_ESM.tif]

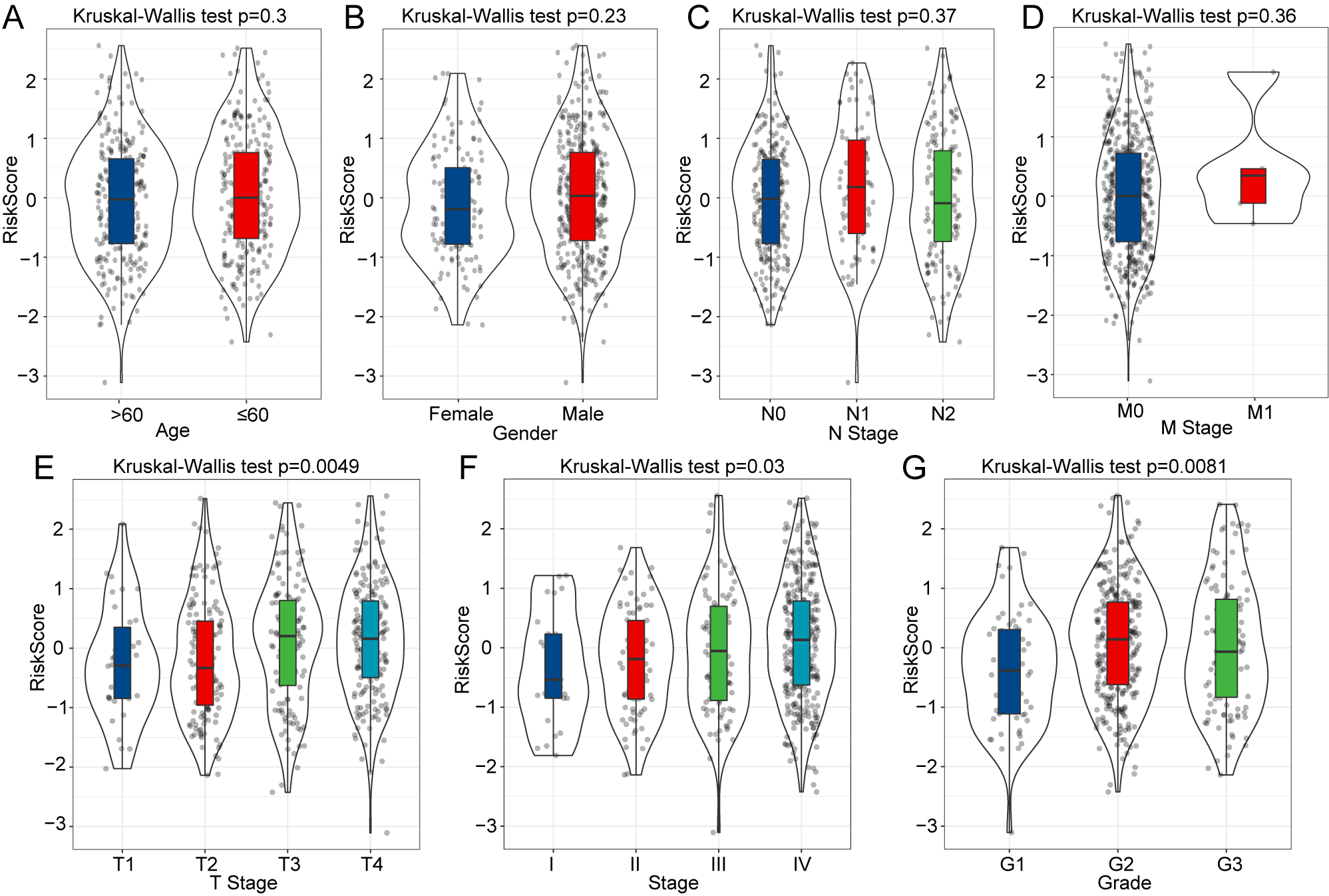

Supplement: Supplementary file 4 — Supplementary Information 4. [file 41598_2022_17898_MOESM4_ESM.tif]
