# Supplementary material for: Construction of a hypoxia-derived gene model to predict the prognosis and therapeutic response of head and neck squamous cell carcinoma
Source: Sci Rep. 2022 Aug 8;12:13538. doi: 10.1038/s41598-022-17898-2 (PMC9363468; doi:10.1038/s41598-022-17898-2)
Supplement: Supplementary file 5 — Supplementary Information 5. [file 41598_2022_17898_MOESM5_ESM.docx]

**Supplementary figure legends**

**Supplementary Figure 1.** Flowchart of our research.

**Supplementary Figure 2.** The proportion of HNSCC patients with HPV-positive and HPV-negative status in three subgroups.

**Supplementary Figure 3.** Protective DEGs and risk DEGs. (**A**) BP annotation map of risk DEGs. (**B**) CC annotation map of risk DEGs. (**C**) MF annotation map of downregulated risk DEGs. (**D**) KEGG annotation map of risk DEGs. Abbreviations: BP, biological process; CC, cellular component; MF, molecular function; KEGG, Kyoto Encyclopedia of Genes and Genomes.

**Supplementary Figure 4.** Excellent prognostic efficacy of the 7-gene signature for patients with different clinical features.
