# Supplementary material for: Construction of a hypoxia-derived gene model to predict the prognosis and therapeutic response of head and neck squamous cell carcinoma
Source: Sci Rep. 2022 Aug 8;12:13538. doi: 10.1038/s41598-022-17898-2 (PMC9363468; doi:10.1038/s41598-022-17898-2)
Supplement: Supplementary file 6 — Supplementary Information 6. [file 41598_2022_17898_MOESM6_ESM.docx]

**Supplementary table 1. Sample information of the TCGA-HNSC, GSE65858, and GSE42743 dataset.**

| **Clinical Features** | **TCGA-HNSC** | **GSE65858** | **GSE42743** |
| --- | --- | --- | --- |
| **OS** |  |  |  |
| 0 | 282 | 176 | 32 |
| 1 | 217 | 94 | 71 |
| **T Stage** |  |  |  |
| T1 | 34 |  |  |
| T2 | 142 |  |  |
| T3 | 132 |  |  |
| T4 | 180 |  |  |
| TX | 11 |  |  |
| **N Stage** |  |  |  |
| N0 | 240 |  |  |
| N1 | 81 |  |  |
| N2 | 152 |  |  |
| N3 | 7 |  |  |
| NX | 19 |  |  |
| **M Stage** |  |  |  |
| M0 | 474 |  |  |
| M1 | 5 |  |  |
| MX | 20 |  |  |
| **Stage** |  |  |  |
| I | 25 | 18 |  |
| II | 80 | 37 |  |
| III | 90 | 37 |  |
| IV | 304 | 178 |  |
| **Grade** |  |  |  |
| G1 | 61 |  |  |
| G2 | 298 |  |  |
| G3 | 119 |  |  |
| G4 | 2 |  |  |
| GX | 19 |  |  |
| **Gender** |  |  |  |
| Male | 366 | 223 | 79 |
| Female | 133 | 47 | 24 |
| **Age** |  |  |  |
| ≤ 60 | 244 | 153 | 54 |
| ＞60 | 255 | 117 | 49 |
