# Supplementary material for: Construction of a hypoxia-derived gene model to predict the prognosis and therapeutic response of head and neck squamous cell carcinoma
Source: Sci Rep. 2022 Aug 8;12:13538. doi: 10.1038/s41598-022-17898-2 (PMC9363468; doi:10.1038/s41598-022-17898-2)
Supplement: Supplementary file 7 — Supplementary Information 7. [file 41598_2022_17898_MOESM7_ESM.docx]

**Supplementary table 2. TCGA training set and verification set sample information table.**

| **Clinical Features** | **TCGA-train** | **TCGA-train** | **P** |
| --- | --- | --- | --- |
| **OS** |  |  |  |
| 0 | 175 | 107 | 0.3086 |
| 1 | 124 | 93 |  |
| **T Stage** |  |  |  |
| T1 | 19 | 15 | 0.6425 |
| T2 | 83 | 59 |  |
| T3 | 75 | 57 |  |
| T4 | 114 | 66 |  |
| TX | 8 | 3 |  |
| **N Stage** |  |  |  |
| N0 | 130 | 110 | 0.1584 |
| N1 | 53 | 28 |  |
| N2 | 98 | 54 |  |
| N3 | 5 | 2 |  |
| NX | 13 | 6 |  |
| **M Stage** |  |  |  |
| M0 | 283 | 191 | 0.894 |
| M1 | 3 | 2 |  |
| MX | 13 | 7 |  |
| **Stage** |  |  |  |
| I | 10 | 15 | 0.12 |
| II | 44 | 36 |  |
| III | 55 | 35 |  |
| IV | 190 | 114 |  |
| **Grade** |  |  |  |
| G1 | 34 | 27 | 0.5369 |
| G2 | 175 | 123 |  |
| G3 | 77 | 42 |  |
| G4 | 2 | 0 |  |
| GX | 11 | 8 |  |
| **Gender** |  |  |  |
| Male | 224 | 142 | 0.3863 |
| Female | 75 | 58 |  |
| **Age** |  |  |  |
| ≤ 60 | 142 | 102 | 0.4984 |
| ＞60 | 157 | 98 |  |
