# Supplementary material for: Construction of a hypoxia-derived gene model to predict the prognosis and therapeutic response of head and neck squamous cell carcinoma
Source: Sci Rep. 2022 Aug 8;12:13538. doi: 10.1038/s41598-022-17898-2 (PMC9363468; doi:10.1038/s41598-022-17898-2)
Supplement: Supplementary file 8 — Supplementary Information 8. [file 41598_2022_17898_MOESM8_ESM.docx]

**Supplementary table 3. The information of primers sequences for qRT-PCR assay.**

| **Primer name** | **Sequence (5'-3')** |
| --- | --- |
| GAPDH-R | GTCATGAGTCCTTCCACGATACC |
| GAPDH-F | GGAGTCCACTGGCGTCTTCA |
| TNFRSF4-R | TCAGTGGGCTGGACAGTGATGG |
| TNFRSF4-F | TCAGAAGTGGGAGTGAGCGGAAG |
| THBS1-R | CATCATCGTGGTCACAGGCATCTC |
| THBS1-F | ATCAGGCAGACACAGACAACAATGG |
| SPINK6-R | TGGGTTAGATTCCCGAGTGC |
| SPINK6-F | AGGCATGTTTCTGCTCCTCTC |
| RNASE7-R | TCGGATGCTTCCCTGAGGTGAG |
| RNASE7-F | CCTTCTGCTGCTTCTGCTGCTG |
| HOXC13-R | CGCTTCTCTTTGGTGATGAACTTGC |
| HOXC13-F | TGGCTGGGACAGTCAGGTGTAC |
| DKK1-R | CACAATCCTGAGGCACAGTCTGATG |
| DKK1-F | CCATTGACAACTACCAGCCGTACC |
| CCL22-R | GAATCATCTTCACCCAGGGCACTC |
| CCL22-F | TGTCCTCGTCCTCCTTGCTGTG |
